# Urinary Spermidine Predicts and Associates with In-Hospital Acute Kidney Injury after Cardiac Surgery

**DOI:** 10.3390/antiox10060896

**Published:** 2021-06-02

**Authors:** Marta Martin-Lorenzo, Angeles Ramos-Barron, Paula Gutierrez-Garcia, Ariadna Martin-Blazquez, Aranzazu Santiago-Hernandez, Emilio Rodrigo Calabia, Carlos Gomez-Alamillo, Gloria Alvarez-Llamas

**Affiliations:** 1 Department of Immunology, Instituto de Investigación Sanitaria-Fundación Jiménez Díaz-UAM, 28040 Madrid, Spain; marta.martin@fjd.es (M.M.-L.); paugugar@gmail.com (P.G.-G.); ariadna.martinb@quironsalud.es (A.M.-B.); aranzazu_sant@hotmail.com (A.S.-H.); 2 Nephrology Department, Hospital Marqués de Valdecilla, IDIVAL, 39008 Santander, Spain; ramosbarron@gmail.com (A.R.-B.); emilio.rodrigo@scsalud.es (E.R.C.); cgalamillo@gmail.com (C.G.-A.); 3 REDInREN, 28040 Madrid, Spain

**Keywords:** acute kidney injury, cardiovascular, KIM-1, NGAL, spermidine, metabolism, oxidative stress

## Abstract

Acute Kidney Injury (AKI) affects up to 30% of the patients who undergo cardiac surgery (CVS) and is related to higher mortality. We aim to investigate molecular features associated with in-hospital AKI development and determine the predictive value of these features when analyzed preoperatively. This is a case-control study. From an initial cohort of 110 recruited subjects, a total of 60 patients undergoing cardiac surgery were included: 20 (33%) developed in-hospital AKI (CVS-AKI) and 40 did not (controls, CVS-C). Pre- and post-surgery samples were collected and a prospective study was carried out. A total of 312 serum samples and 258 urine samples were analyzed by nuclear magnetic resonance, mass spectrometry and ELISA. Six features predicted AKI development in pre-surgery samples: urinary kidney functional loss marker kidney injury molecule-1 (uKIM-1), 2-hydroxybutyric acid, 2-hydroxyphenylacetic acid, hippuric acid, phosphoethanolamine and spermidine. Two of them stood out as powerful predictors. Pre-surgery uKIM-1 levels were increased in CVS-AKI vs. CVS-C (AUC = 0.721, *p*-value = 0.0392) and associated strongly with the outcome (OR = 5.333, *p*-value = 0.0264). Spermidine showed higher concentration in CVS-AKI (*p*-value < 0.0001, AUC = 0.970) and had a strong association with the outcome (OR = 69.75, *p*-value < 0.0001). uKIM-1 and particularly spermidine predict in-hospital AKI associated with CVS in preoperative samples. These findings may aid in preventing postoperative AKI and improve prognosis of CVS.

## 1. Introduction

Acute Kidney Injury (AKI) is a serious postoperative complication affecting up to 30% of cardiac surgery patients [1]. It is associated with higher morbidity and mortality [2,3,4] and increased economic burden for healthcare systems [5]. The effects of in-hospital AKI have been related to serious long-term consequences such as shorter life span and chronic dialysis [6]. However, it is not possible to anticipate AKI development in the current clinical practice. AKI patient management usually consists of additional surgery to preserve kidney functionality, although the outcome is not always satisfactory [7].

In recent years, there has been a growing interest in the early prevention of post-surgery AKI, with particular emphasis on finding novel markers that contribute to early detection. Research has mainly been focused on kidney tubular damage indicators such as urinary neutrophil gelatinase-associated lipocalin (uNGAL) and kidney injury molecule-1 (uKIM-1), in addition to markers of kidney function such as serum cystatin C (sCysC). These markers have shown promising results for an early post-surgery detection, especially when combined [8]. Additionally, markers of cardiac function and inflammation have increased understanding of the disease but have yet to demonstrate good prediction capacity [9,10,11].

Although useful, postoperative prediction is still too late. The gold standard for AKI diagnosis, serum creatinine (sCr), is not a predictor but rather an indicator of established renal damage. Additionally, AKI develops shortly after surgery (usually within the first 6h), which gives physicians a very narrow window in which to act. As a result, more research is needed to identify molecular predictors that can be used to evaluate risk of post-surgery AKI when analyzed in pre-surgery samples.

The usefulness of metabolites as potential predictors of in-hospital AKI has shown promising results in plasma samples collected at 24 h post-surgery [12]. Our group identified a set of urinary metabolites involved in renal disease, which do not only reflect the evolution AKI but also severity of injury, distinguishing between acute tubular necrosis AKI and prerenal AKI [13].

In the present study, we aimed to investigate molecular features that can predict in-hospital AKI after CVS, even before surgery. For such purpose, known markers of tubular damage and kidney dysfunction, together with a novel metabolite panel here identified in association with post-surgery AKI, were investigated in pre-surgery urine to predict the outcome. The post-surgery variation of those molecules was also evaluated over time.

## 2. Materials and Methods

### 2.1. Study Population, Clinical Data, and Sample Collection

A total of 110 subjects with cardiovascular disease who were admitted for cardiac surgery at University Hospital Marqués de Valdecilla in Santander were considered for this observational study. Inclusion criteria were patients of both sex and ages between 18 and 85 years who underwent extracorporeal cardiac surgery of any etiology. All patients were subjected to previous catheterization. Exclusion criteria were patients with active immunological disease under treatment, visceral neoplastic disease within the last 5 years patients on chronic dialysis (hemodialysis or peritoneal dialysis), patients with solid organ transplant and patients with an expected survival of less than one year. Patients fulfilling the inclusion and exclusion criteria who consented to participate in the study were prospectively studied. All patients were informed that their participation in the study would not jeopardize the treatment in any way or put them at risk and signed the informed consent form. The study was conducted according to the recommendations of the Declaration of Helsinki and was approved by local ethics committee (PIC181-19).

Analytical and clinical data were collected from clinical records and electronic databases of the hospital and primary care, including patient characteristics and comorbidities. Urine and serum samples were collected from patients at different time points during admission: pre-surgery (P), followed by 6, 24, 48 and 72 h post-surgery and at discharge (D), resulting in a total of 312 serum samples and 258 urine samples (collection of sufficient amount of urine was not possible in all cases). Serum was obtained from blood samples by centrifugation at 3000× *g* for 10 min. Similarly, urine samples were centrifuged at 1300× *g* for 5 min to remove cell debris. Processed samples were frozen and stored at −80 °C until analysis.

### 2.2. Clinical Outcome Definitions

The primary outcome of this study was in-hospital post-surgical AKI defined by the Kidney Disease: Improving Global Outcomes (KDIGO) guidelines as an increase in serum Creatinine (sCr) concentration of ≥0.3 mg/dL within 48 h after surgery [14]. Creatinine basal levels were determined in a serum sample collected the day before the surgery, which was considered as the basal situation of the patient. Accordingly, patients were included in the control group (CVS-C) if their sCr levels (mg/dL) showed no significant increase in sCr (*n* = 40), or in the AKI group (CVS-AKI) (*n* = 20) if the increment in sCr was ≥0.3 mg/dL. In all cases, CVS-AKI patients had sCr levels corresponding to stage 1 of the AKI KDIGO classification.

### 2.3. Metabolic Screening of In-Hospital Kidney Injury

An initial screening analysis to identify altered metabolites in patients who developed post-surgery AKI was performed by ^1^H Nuclear Magnetic Resonance (NMR) as described before [13,15,16,17,18,19]. In this first metabolomics screening, no molecular candidates to be analyzed were selected beforehand. Potential candidates from NMR analysis were then quantitatively analyzed by targeted mass spectrometry in selected reaction monitoring mode (SRM) as later described. For NMR analysis, trimethylsilyl propionate was added to all samples for chemical shift referencing and NMR experiments were performed at 277 K on a Bruker 700 MHz AVANCE III instrument. Spectra were processed using TOPSPIN (version 1.3, Bruker Biospin Ltd., Rheinstetten, Germany) and statistically analyzed using AMIX software (version 3.6.8, Bruker, Rheinstetten, Germany). Each spectrum was partitioned into small spectral regions of 0.02 and 0.04 ppm (buckets) that were pre-processed by unit variance and pareto scaling. Considering 95% confidence, significant buckets were identified as those with *p* < 0.05 for further investigation. Bucket-to-metabolite conversion was performed using the Metabohunter tool [20] and Human Metabolome Database (HMDB). Two-dimensional NMR analysis, including homonuclear correlation spectroscopy ^1^H–^1^H (COSY), total correlated spectroscopy (TOCSY) and heteronuclear single-quantum correlation spectroscopy (^1^H–^13^C HSQC), were used for confirmation.

### 2.4. Quantification of Urinary Metabolites Pre- and Post-Surgery. Identification of Metabolic Markers of Renal injury

Urine samples were centrifuged at 16,200× *g* for 15 min at 4 °C and metabolite extraction was performed with acidified acetonitrile (0.1% formic acid). Metabolite quantitation was performed by liquid chromatography and mass spectrometry in tandem (LC–MS/MS) in selected reaction monitoring mode (SRM). Concentration values were calculated by interpolation in calibration curves performed with commercial standards. A 6460 Triple Quadrupole coupled to HPLC1200 Series (Agilent Technologies, Waldbronn, Germany) was used, controlled by Mass Hunter Software (v4.0, Agilent Technologies, Waldbronn, Germany). Separation took place at 0.4 mL/min in an acetonitrile gradient for 5 min in positive or negative mode. The optimal analytical conditions are summarized in Supplementary Table S1 and were previously set up with commercial standards. The concentration ranges used for the different metabolite determinations were as follows: 0–8.4 μg/mL for 2-hydroxybutyric acid; 0–12 ng/mL for 2-hydroxyphenylacetic acid; 0–12 μg/mL for hippuric acid; 0–0.28 μg/mL for N-acetylneuraminic acid; 0–0.06 μg/mL for pantothenic acid; 0–15 ng/mL for phosphoethanolamine; 0–0.17 μg/mL for spermidine; and 0–1.2 μg/mL for succinic acid. Urinary creatinine concentration was determined as described elsewhere [21]. Final metabolite concentration was normalized by urinary creatinine, resulting in a final concentration value expressed as μM/mM Creatinine or nM/mM Creatinine.

### 2.5. Analysis of Protein Markers of Renal Tubular Damage

Serum and urine samples were thawed for batch analysis of renal tubular injury protein markers sCysC, uNGAL and uKIM-1. sCysC was measured as described before without modifications [21]. sCysC concentration is expressed in mg/L. uNGAL quantification was estimated by Enzyme-linked immunosorbent assays (ELISA) following manufacturer recommendations (Bioporto Diagnosis, Hellerup, Denmark). Briefly, urine samples were centrifuged at 600× *g* for 10 min, diluted, and measured in a range of 10–1000 pg/mL. Similarly, uKIM-1 quantitation was determined by ELISA (Enzolife science, Farmingdale, NY, USA). Samples were centrifuged (600× *g*, 10 min), diluted, and evaluated in a concentration range (0–500 pg/mL). Final concentration values for uNGAL and uKIM-1 were normalized by urinary creatinine concentration and expressed as ng/mg creatinine.

### 2.6. Statistical Analysis

Statistical analysis was performed using GraphPad Prism 8.0.2 (GraphPad Software, Inc., San Diego, CA, USA). The ROUT method was applied to detect outliers based on the false discovery rate, setting the Q value to 5%. Mann–Whitney non-parametric test (two-tailed) was performed. In the case of multiple analyses, the Kruskal–Wallis test was applied. Correlation of the molecular features of interest with sCr and estimated glomerular filtration rate (eGFR) was determined by a Spearman non-parametric test. The influence of the co-factors male sex, age, arterial hypertension, diabetes mellitus, eGFR and chronic kidney disease (CKD) on the prediction capacity of molecules was evaluated by linear regression in R (version 1.1.442).

Individual and combined prediction capacity was evaluated by receiving operating characteristics (ROC) curves using Metabonalyst [22] for the pre-surgery samples. The area under the curve (AUC) was obtained and the value closest to the top left corner was considered as cut-off value for biomarker evaluation. By two by two tables and logistic regression in Epi Info 7.2.4 and GraphPad Prism 8.0.2., associations between feature concentrations and the primary outcome were estimated and expressed as odds ratio (OR) (95% confidence interval) with a two-tailed p-value statistical analysis. For those features predicting the outcome and with a strong association (OR > 3.5), adjusted ORs by eGFR and uKIM-1 were estimated. Unadjusted OR calculation was performed following cross product and the 95% confident intervals were estimated based on Taylor series. The adjusted OR was based on a logistic regression model.

## 3. Results

### 3.1. Clinical Characteristics

Among an initial cohort of 110 subjects, 22 surgeries were postponed resulting in 88 patients. It was not possible to assure proper sample recruitment for 25 of them so they were dismissed and three more retired their consent during the study, resulting in a final study cohort of 60 patients. Forty patients (67%) did not develop in-hospital AKI following cardiac surgery and constituted the control group (CVS-C); the other 20 patients (33%) had an increase in sCr levels of over 0.3 mg/dL during the first 48 h and were thus assigned to the in-hospital AKI group (CVS-AKI). Table 1 contains the baseline patients’ clinical characteristics (pre-surgery) and data on markers of renal function at 24 h post-surgery. In view of previous evidence showing oxidative stress is a known injury mechanism associated to AKI, cardiovascular risk factors as hypertension, diabetes mellitus, cerebrovascular accident, AMI, PAD, or CKD were considered as comorbidities, which may influence a higher incidence of suffering post-surgical AKI.

Both groups had similar demographic characteristics in terms of age, gender, weight, and body mass index (BMI); these patients had not significant differences in their lipid profile or comorbidities, but peripheral artery disease. CVS-AKI subjects had a higher Charlson comorbidity index and a higher incidence of valvular surgery (90%). Renal function based on sCr, eGFR and urine output showed no differences between clinical groups at baseline (pre-surgery).

### 3.2. Altered Urine Metabolites in AKI vs. Non-AKI Patients

The screening analysis conducted using NMR resulted in a total of 55 spectral features (buckets) that discriminated between CVS-C and CVS-AKI patients (Supplementary Table S2). Statistically significant buckets correspond to eight metabolites: 2-hydroxybutyric acid, 2-hydroxyphenylacetic acid, hippuric acid, *N*-acetylneuraminic acid, pantothenic acid, phosphoethanolamine, spermidine and succinic acid (Supplementary Table S3). All these metabolites were further analyzed by mass spectrometry-based absolute quantitation (targeted approach). Supplementary Figure S1 shows the calibration curves used to calculate metabolite concentrations. Phosphoethanolamine was the least abundant metabolite in urine for both CVS-C and CVS-AKI patients, with concentrations in the range of 0.1–100 nM/mM creatinine, which was close to the detection limit. In contrast, hippuric acid was in the most abundant metabolite with concentration values in the range of 1–45 μM/mM creatinine. The exact concentration values at the different time points studied (P, 6 h, 24 h, 48 h, 72 h and D) are detailed in Supplementary Table S4.

### 3.3. Surgery Is Reflected in the Metabolic Urinary Profile Independently of AKI

**Figure 1:**
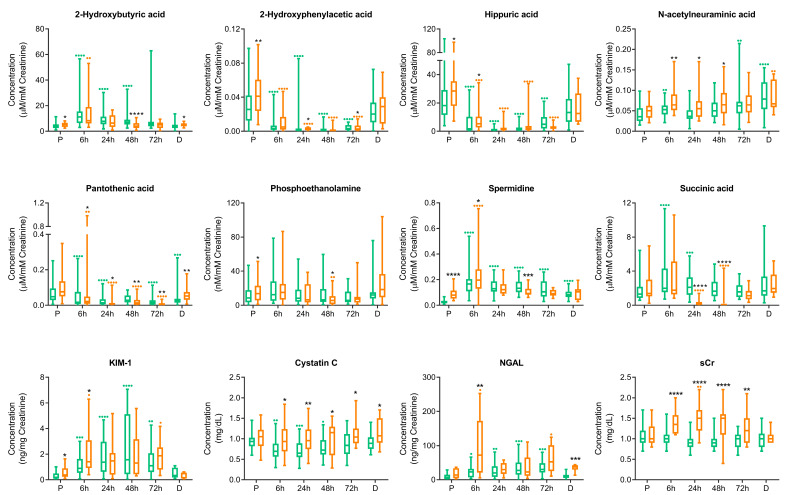
Concentration values variation over time of molecular features identified in association with post-surgery AKI. Comparison of levels between CVS-C and CVS-AKI subjects is represented in boxplot (min–max) at each point of time (pre-surgery: P; post-surgery: 6, 24, 48, 72 h and discharge (D)). Concentration levels over time of CVS-C subjects are shown in green and levels in CVS-AKI subjects are shown in orange. Mann–Whitney statistical test was performed to compare clinical groups at each timepoint (CVS-C vs. CVS-AKI): * *p* < 0.05, ** *p* < 0.01, *** *p* < 0.001, **** *p* < 0.0001 (significance in black). The Kruskal–Wallis statistical test was performed to identify significant variations between post-surgery points of time (6, 24, 48, 72 h or D) and pre-surgery levels (P): • *p* < 0.05, •• *p* < 0.01, ••• *p* < 0.001, •••• *p* < 0.0001 (significance in green for CVS-C values and in orange for CVS-AKI values).

As can be seen in Figure 1, all metabolites except phosphoethanolamine showed an early alteration just after surgery (6 h). In the case of 2-hydroxybutyric acid, 2-hydroxyphenylacetic acid, hippuric acid, pantothenic acid and spermidine, this alteration was observed for both CVS-C and CVS-AKI patients. 2-Hydroxyphenylacetic acid and hippuric acid maintained significant alteration from pre-surgery levels to 72 h normalizing at discharge. Other urinary metabolites did not seem to be as clearly influenced by surgery, as in the case for phosphoethanolamine. The urinary metabolites abundance may vary along time in response to the surgery itself. Where a difference in urinary metabolite abundance is observed in response to AKI, surgery-related variation over time should be evaluated for the CVS-C group. For CVS-AKI subjects, overlapping responses can be observed. This is the case for pantothenic acid, succinic acid and spermidine. Hippuric acid, 2-hydroxyphenylacetic acid and pantothenic acid showed similar alterations over time in CVS-C and CVS-AKI, thus indicating an alteration mainly due to surgery. Conversely, a differential effect is shown for 2-hydroxybutyric acid and spermidine, which seem to modulate their concentrations in CVS-C patients as compared to baseline, at which point a different pattern is seen in CVS-C and CVS-AKI patients.

Regarding differences between CVS-AKI and CVS-C groups attributed to AKI, hippuric acid, N-acetylneuraminic acid, pantothenic acid and spermidine showed early alterations just after surgery (6 h), and N-acetylneuraminic acid and pantothenic acid maintained an altered state at 48 and 72 h. Conversely, 2-hydroxybutyric acid and phosphoethanolamine only presented statistically significant alterations 48 h after surgery, indicating a late response.

### 3.4. sCr, sCysC, uNGAL and uKIM-1 Variation after Cardiovascular Surgery in AKI and Non-AKI Patients

An evaluation of protein markers of renal injury and sCr was performed at the different post-operative time points. As expected, sCr clearly distinguished between CVS-C and CVS-AKI patients during in-hospital stay, as of 6 h until discharge, and was not influenced by surgery itself (Figure 1). In contrast, surgery influenced the levels of renal damage markers sCysC, uNGAL and uKIM-1 starting at 6 h post-surgery (i.e., AKI-C trends along time). In the case of sCys, its levels normalize at 48 h; uKIM-1 and uNGAL do at 72 h.

Additionally, the three protein markers distinguished AKI from non-AKI patients at 6 h, with an increasing trend for CVS-AKI group compared to CVS-C group. uKIM-1 only discriminated between both clinical groups at 6 h and uNGAL results were more random. sCysC remains significantly elevated in CVS-AKI along time, even at discharge.

### 3.5. Molecular Marker Correlation with sCr and eGFR

To evaluate the renal component of the molecular alterations identified, correlations were evaluated between the proteins and metabolites studied and sCr levels and eGFR. N-acetylneuraminic acid, 2-hydroxybutyric acid, succinic acid, sCysC and uNGAL correlated with both, sCr and eGFR. Pantothenic acid correlated exclusively with sCr levels. Those presenting a positive correlation with sCr were uNGAL, sCysC and N-acetylneuraminic acid. Negative correlations were found for 2-hydroxybutyric acid, pantothenic acid and succinic acid. All of them but pantothenic acid correlated with eGFR, showing the opposite trend as that observed with sCr. uKIM-1, 2-hydroxyphenlacetic acid, hippuric acid, phosphoethanolamine and spermidine did not correlate with sCr or eGFR (Table 2).

### 3.6. uKIM-1 and Spermidine Predict AKI Development after Cardiovascular Surgery

Of special interest in clinical practice is the identification of predictors of postoperative in-hospital AKI, ideally before surgery takes place. With this aim, analysis of the modulated molecules previously described was carried out in pre-surgical samples to evaluate their ability to predict AKI development. sCr, sCysC and uNGAL did not show significantly different concentrations between AKI and non-AKI patients before surgery (data not shown). The three of them can be used to monitor renal damage in our cohort; however, they failed to predict the outcome. In contrast, uKIM-1 showed significantly increased levels in CVS-AKI vs. CVS-C in pre-surgical urinary samples and thus has potential predictive value.

**Figure 2:**
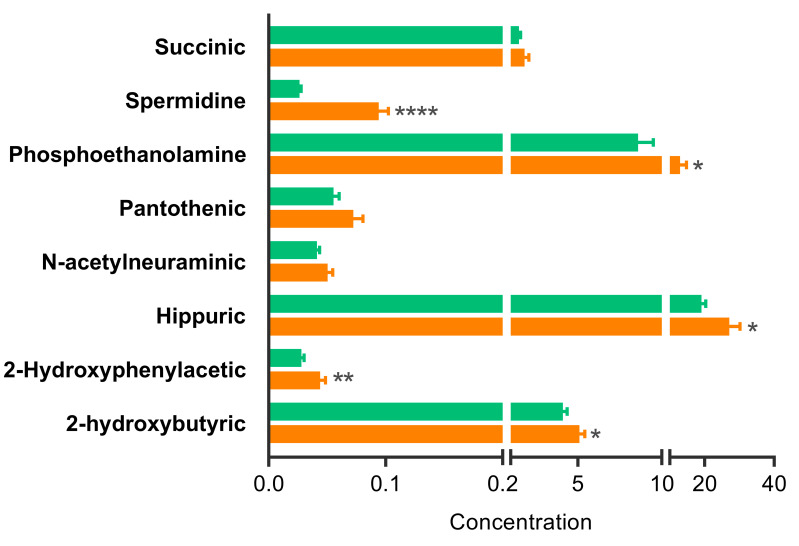
Differential analysis of metabolite concentrations in pre-surgery urine samples. Concentration values are expressed in uM/mM Creatinine for all metabolites except phosphoethanolamine, for which the concentration value is expressed in nM/mMCreatinine. Green bars: CVS-C; Orange bars: CVS-AKI. Mann–Whitney: * *p* < 0.05, ** *p* < 0.01, **** *p* < 0.0001.

Regarding to the metabolites, 2-hydroxybutyric acid, 2-hydroxyphenylacetic acid, hippuric acid, phosphoethanolamine and spermidine showed altered concentrations before surgery between CVS-C and CVS-AKI, indicating its ability to predict in-hospital AKI development. Figure 2 shows the difference in concentration for these metabolites prior to surgery together with their statistical significance.

The predictive value of these molecular markers of renal damage, based on concentration level, was evaluated by calculating ROC curves (see Supplementary Figure S2). A modest ability to predict AKI development was found for most of the metabolites, proteins and clinical parameters evaluated (AUC value around 0.6) with the exception of uKIM-1 and spermidine with AUC values of 0.7214 and 0.9700, respectively. The prediction capacity remains significant even when adjusted by cardio-renal risk co-factors such as age, sex, arterial hypertension, diabetes mellitus, eGFR or CKD (data not shown). Spermidine thus represents the best marker candidate for predicting postoperative AKI complications in cardiovascular disease patients.

### 3.7. Pre-Surgery Concentrations of Urinary Predictors Associate with AKI Outcome

To quantify the potential association between molecular concentrations in pre-surgical samples and the outcome (post-surgery AKI), logistic regression analysis was performed and ORs were estimated (Table 3). With the exception of uNGAL, N-acetylneuraminic acid and succinic acid, all the molecules presented a positive significant association with the outcome. Most of the ORs values ranged from 2.5 to 5.5. Spermidine, with an OR of 69.75 (*p* > 0.0001), showed the strongest association with the outcome in our cohort (cut-off value = 0.0455 µg/mg Creatinine, Supplementary Figure S2).

uKIM-1, Spermidine, sCysC and pantothenic acid showed the highest associations with the outcome. When ORs were adjusted by eGFR as renal function indicator, uKIM-1 and spermidine slightly decreased their association with postoperative AKI, though the association for pantothenic acid increased (from OR= 3.500 to OR= 5.769). When the ORs were adjusted by uKIM-1, both increased their association with the outcome, and spermidine adjusted OR increased to 158.1 (*p* = 0.0001).

## 4. Discussion

We show here a multiple analysis of features associated with in-hospital AKI after cardiac surgery, some with potential to predict AKI development based on pre-surgery samples data. Known AKI-related features and novel metabolites not previously associated with AKI were analyzed and compared. Their postoperative modulation with time and their predictive value in pre-surgery urine samples was investigated to identify early markers. The characterization of early markers in pre-surgery samples is underexplored, as most existing studies have focused on post-surgery analysis within the first 6h. This difference with respect to the previous literature makes our study unique and of interest.

In this study, patients with higher Charlson comorbidity score, PAD or valvular surgery showed higher rate of post-surgical AKI in agreement with previous studies [23,24], without relation to other cardiovascular comorbidities, cardiovascular risk factors, by-pass time or ICU stay length. AKI after cardiac surgery has a multifactorial pathogenesis that can be related to processes such as renal fluid depletion, dysfunctional inflammatory cascades, oxidative stress, activation of proapoptotic pathways, changes in molecular expression, and leukocyte trafficking, among others [25]. Inflammation molecules, cardiac surgery molecular alterations, renal injury and renal tubular damage-associated markers have been proposed as molecular indicators of AKI [7].

uKIM-1 is a known marker of renal injury. It is a transmembrane glycoprotein produced by the tubular cells and it is known to be released into urine following ischemic tubular damage [7]. We identified increased levels of uKIM-1 for both CVS-C and CVS-AKI subjects when comparing pre- and postoperative samples. The increase in uKIM-1 levels remained during the hospital stay and normalized to pre-surgery levels at discharge, thus reflecting reversible kidney damage caused by surgery. uKIM-1 has been associated with early post-surgery detection of AKI associated with cardiac surgery [8,26,27]. The results from our cohort support these earlier findings, showing significantly different concentrations between CVS-C and CVS-AKI patients as early as 6h. Furthermore, pre-surgery uKIM-1 levels were significantly increased in patients developing AKI, which means that this marker has predictive value even before surgery. In this sense, uKIM-1 was associated with the primary outcome and subjects undergoing cardiac surgery with increased levels of uKIM-1 are five times more likely to develop in-hospital AKI independently of their baseline eGFR and sCr levels.

The alteration of metabolites levels in urine not only reflects pathophysiological responses to a surgical trauma, but rather post-surgery AKI influences and may even predispose CVS patients to this renal complication. Interestingly, all metabolites that have potential to predict AKI development based on pre-surgery samples, with the exception of 2-hydroxybutyric acid, did not correlate either with sCr or eGFR, pointing to mechanisms other than renal damage. Conversely, other metabolites such as N-acetylneuraminic acid, pantothenic acid or succinic acid do not predict AKI in pre-surgical samples, but correlate with sCr and/or inversely with eGFR, clearly reflecting kidney injury.

Of all the metabolites investigated, spermidine showed the strongest capacity to anticipate AKI even in preoperative samples. Spermidine is considered the most biologically active polyamine and its concentration is partly controlled by renal secretion [28]. It has been argued to play a role in CKD etiology, since it has a strong association with longitudinal eGFR decline [29]. We found no correlation with the eGFR or with sCr but a strong association between spermidine and the outcome was observed. Patients with a preoperative urine spermidine of higher than 0.0455 µM/mM Creatinine are 70 times more likely to develop in-hospital AKI than those with lower concentrations. If the association is adjusted by uKIM-1, the adjusted OR increases up to 158. Regarding its predictive value, spermidine itself strongly distinguish between clinical groups with an AUC of 0.97. Furthermore, spermidine levels remained significantly altered 6 h after surgery and thus it could be also considered a postoperative marker of CVS-related AKI. Spermidine is a natural polyamine with anti-inflammatory properties, antioxidant effects and enhancement of mitochondrial metabolic function [28]. Spermidine beneficial role was shown in the kidney by ameliorating renal function decline in animal models of aging and hypertension [30,31], and by inhibiting renal oxidative stress after ischemia reperfusion injury [32,33]. However, spermidine targets are usually the cardiac and vascular systems. Altered polyamine metabolism contributes to an increase in oxidative stress and tissue damage and particularly spermidine is a key player of the oxidative balance of the cells [34]. Here, we observed increased levels in urine, which may indicate reduced cellular levels resulting from higher renal excretion. This potential depletion of cellular spermidine may in turn result in a reduced capacity to counteract oxidative stress and thus induced kidney damage [35]. On the other hand, oxidative stress is a known injury mechanism associated to AKI [24,36,37], thus increased pre-surgery levels of spermidine may be a reflection of an enhanced baseline oxidative status which, added to the oxidative stress caused by cardiac surgery per se, may result in higher predisposition to develop AKI.

Urinary spermidine may help identify early those patients at high risk of developing AKI after cardiac surgery. Using a similar approach, an early identification of “high risk AKI patients” by using a combination of biomarkers such as [TIMP-2]·[IGFBP7] reduced the risk of AKI related to cardiac surgery from 71.7% to 55.1% (*p* = 0.004) through an strictly controlled implementation of the KDIGO guidelines, avoiding nephrotoxic agents and radiocontrast agents, discontinuing ACEi and ARBs, monitoring serum creatinine and urine output closely, avoiding hyperglycemia and optimizing the volume status and hemodynamic parameters by using a PICCO catheter and a pre-specified algorithm [38].

The present study fulfills the requirements of an omics study in terms of group size and technical workflow; however, one limitation could be the relatively low number of patients from a clinical perspective. To overcome this, two phases were used, screening and confirmation by absolute quantitation. Further studies are warranted in larger cohorts before considering the use of these new urinary biomarkers in clinical practice.

## 5. Conclusions

The urinary metabolome reflects the effects of CVS and predicts AKI outcome. In pre-surgery urine samples, specific metabolites and tubular damage proteins may anticipate AKI development. Particularly, urinary spermidine significantly correlates with and predicts AKI after cardiac surgery in preoperative stage, thus allowing very early prediction of the outcome. Spermidine is a polyamine with known antioxidant properties and the most significant alteration found in pre-surgery urine of patients developing post-surgical AKI. This study gives evidence of a potential imbalance in the oxidative status of these patients, encouraging novel research in pre-operative antioxidant treatments as new therapeutic avenues for AKI prevention. This knowledge may be used to bring substantial improvements to patient management and the prognosis of patients with cardiovascular disease.

## Figures and Tables

**Table 1:** Baseline characteristics (pre-surgery) and markers of renal function at 24 h post-surgery of patients included in the study.

Baseline Characteristics (Pre-Surgery)	CVS-C	CVS-AKI	*p*-Value
Number of patients	40	20	
Age, years (†)	68 ± 12	73 ± 8	0.097
Gender (males) (*)	30 (75)	11 (55)	0.116
Comorbidities (*)			
Hypertension	32 (80)	16 (80)	1
Diabetes mellitus	7 (17.5)	8 (40)	0.058
Cerebrovascular accident	2 (5)	0 (0)	0.309
AMI	9 (22.5)	3 (15)	0.494
Peripheral vascular disease	13 (32.5)	15 (75)	0.002
Chronic kidney disease	6 (15)	7 (35)	0.076
Congestive heart failure	8 (20)	7 (35)	0.206
Endocarditis	1 (2.6)	0	0.470
PAH	2 (5.1)	4 (20)	0.074
Charlson comorbidity score (†)	4.08 ± 2.08	5.85 ± 1.98	0.002
Weight (kg) (†)	80 ± 11	76 ± 11	0.217
BMI (kg/m^2^) (†)	29 ± 3	29 ± 3	0.687
Total cholesterol (mg/dL) (†)	178 ± 36	180 ± 31	0.872
LDL cholesterol (mg/dL) (†)	108 ± 29	102 ± 26	0.510
HDL cholesterol (mg/dL) (†)	46 ± 13	55 ± 24	0.129
Triglycerides (mg/dL) (†)	120 ± 43	113 ± 57	0.661
Previous hyperlipidemia (*)	25 (71)	16 (80)	0.483
Previous hyperlipidemia treatment (*)	27 (73)	16 (80)	0.556
Nephrotoxic drugs (≥1) (*)	36 (90)	16 (80)	0.283
Previous angiography (*)	29 (72.5)	17 (85)	0.281
Type of surgery			
Only valvular (*)	25 (62.5)	18 (90)	0.026
Only coronary(*)	16 (40)	8 (40)	1
ByPass (*)	38 (95)	19 (95)	1
Ischemia time (min) (†)	79 ± 28	90 ± 39	0.252
CPB time (min) (†)	105 ± 34	120 ± 50	0.166
Left ventricular ejection fraction (%) (**)	60 (10)	60 (10)	1
ICU stay, days (**)	2 (2)	2 (3)	0.927
Markers of renal function			
sCreatinine (mg/dL) (†)	1.05 ± 0.23	1.12 ± 0.28	0.325
eGFR (CKD-EPI) (†)	71 ± 18	61 ± 19	0.070
eGFR (MDRD) (†)	73 ± 17	64 ± 20	0.096
Urine output* (≤400 mL/24 h)	0 (0)	0 (0)	1
**Markers of Renal Function** **at 24 h Post-Surgery**	**CVS-C**	**CVS-AKI**	***p*-Value**
KDIGO *			0.000
0	40 (100)	5 (25)	
1	0	15 (75)	
sCreatinine (†)	0.98 ± 0.24	1.49 ± 0.36	0.000
eGFR (†)	77 ± 20	44 ± 15	0.000
Urine output * (≤400 mL/24 h)	3 (7.5)	0 (0)	0.209

Values are expressed as * number (percent), † mean ± SD, or ** median (range). AMI: Acute myocardial infarction; PAH: Pulmonary arterial hypertension; BMI: Body mass index; LDL: low-density lipoprotein; HDL: high-density lipoprotein; CPB: cardio pulmonary bypass; ICU: intensive care unit; eGFR: estimated glomerular filtrate rate (by Chronic Kidney Disease Epidemiology Collaboration (CKD-EPI)). AKI was defined and classified according KDIGO criteria.

**Table 2:** Spearman correlation values for metabolites and proteins with sCr and eGFR are presented.

	sCr (mg/dL)		eGFR (mL/min)	
	*r*	*p*-Value	*r*	*p*-Value
**AKI Post-CVS Metabolic Markers**				
2-hydroxybutyric acid	−0.272	**<0.001**	0.2151	**<0.001**
2-hydroxyphenylacetic acid	−0.01721	0.80	−0.02630	0.70
Hippuric acid	0.03536	0.57	−0.1050	0.10
N-acetylneuraminic acid	0.2193	**<0.001**	−0.3373	**<0.001**
Pantothenic acid	−0.1391	**0.03**	0.09505	0.14
Phosphoethanolamine	−0.001941	0.98	−0.01219	0.85
Spermidine	−0.04087	0.52	0.004941	0.94
Succinic acid	−0.3313	**<0.001**	0.2447	**<0.001**
**Renal Functional Markers**				
Cystatin C	0.5847	**<0.001**	−0.6126	**<0.001**
uNGAL	0.2964	**<0.001**	−0.2407	**<0.001**
uKIM-1	0.04008	0.54	0.06392	0.33

**Table 3:** Logistic regression evaluation for the association of independent features with in-hospital AKI outcome.

		OR [95% CI]	*p*-Value
**Renal Functional Markers**			
	eGFR	0.4582 [0.1529–1.372]	0.18
	sCysC	4.714 [1.317–16.87]	0.02
	uKIM-1	5.333 [1.226–23.20]	0.03
	uNGAL	3.330 [0.9174–12.11]	0.11
**AKI Post-CVS Surgery Metabolic Markers**			
	2-hydroxybutyric acid	3.302 [1.349–8.084]	0.01
	2-hydroxyphenylacetic acid	2.623 [1.008–6.370]	0.047
	Hippuric acid	3.302 [1.349–8.084]	0.01
	N-acetylneuraminic acid	2.178 [0.8944–5.302]	0.12
	Pantothenic acid	3.500 [1.401–8.744]	0.008
	Phosphoethanolamine	2.521 [0.9877–6.434]	0.07
	Spermidine	69.75 [17.17–283.4]	<0.001
	Succinic acid	2.000 [0.8167–4.898]	0.19
**Adjusted Best Predictor Features by eGFR**			
	Spermidine	65.83 [9.312–465.4]	<0.001
	Pantothenic acid	5.769 [1.267–26.25]	0.02
	uKIM-1	4.552 [0.9980–20.76]	0.05
**Adjusted Best Predictor Features by uKIM-1**			
	Spermidine	158.1 [11.97–2088]	0.0001
	Pantothenic acid	5.454 [1.251–23.77]	0.02

## Data Availability

The datasets used and/or analyzed during the current study are included in this published article and its supplementary information files.

## Supplementary Materials

The following are available online at https://www.mdpi.com/article/10.3390/antiox10060896/s1, Table S1: Mass Spectrometry conditions for target analysis (SRM-LC-MS/MS). Table S2: Statistically significant NMR bucket shifts for CVS-C vs. CVS-AKI comparison. Table S3: NMR identified metabolites. Table S4: Experimental concentration of identified metabolites at different times from pre-surgery (P) to discharge (D). Concentration values are expressed in µM/mMCreatinine except for Phosphoehtanolamine whose concentration is shown as nM/mMCreatinine. *p*-value is the result of non-parametric Mann-Withney test between CVS-C and CVS-AKI patient. Bold letters correspond to statistically significant differences (*p* < 0.05). Figure S1: Metabolites were quantified by SRM-LC-MS/MS. Equations for the lineal fit of each metabolite (or fit applied), concentration ranges and the square of the coefficient values (R2) are shown. Figure S2: Individual Receiving Operating Curves (ROC) for Metabolites, Proteins and Clinical parameters. The images show the Area Under the Curve (AUC) and the optimal cutoff at closest to top-left corner values. Both expressed with 95% IC.
