# Supplementary material for: Urinary Spermidine Predicts and Associates with In-Hospital Acute Kidney Injury after Cardiac Surgery
Source: Antioxidants (Basel). 2021 Jun 2;10(6):896. doi: 10.3390/antiox10060896 (PMC8229689; doi:10.3390/antiox10060896)
Supplement: Supplementary file 1 [file antioxidants-10-00896-s001.zip › antioxidants-1247214-supplementary.pdf]

Supplementary Table S1. Mass Spectrometry conditions for target analysis (SRM-LC-MS/MS)

**METABOLITE**	**SRM Transition** m/z precursor ion → m/z fragment ion	**Fragmentor** (V)	**CE** (V)	**Polarity**
2-Hydroxybutyric acid	103.1 → 57.1	120	10	Negative
2-Hydroxyphenylacetic acid	151.2 → 107.1	72	10	Negative
Hippuric acid	180.2 → 105.0	80	14	Positive
N-Acetylneuraminic acid	308.3 → 87.0	90	10	Negative
Panthotenic acid	220.2 → 90.1	80	14	Positive
Phosphoethanolamine	142.1 → 44.3	80	10	Positive
Spermidine	146.3 → 72.2	60	14	Positive
Succinic	117.1 → 73.2	70	10	Negative

Supplementary Table S2. Statistically significant NMR bucket shifts for CVS-C *vs.* CVS-AKI comparison.

δ (ppm)	p-value
1.28	0.014
1.49	0.010
1.63	0.043
2.35	0.048
2.52	0.020
2.55	0.021
2.68	0.047
2.69	0.014
2.70	0.0019
2.91	0.031
3.16	0.014
3.18	0.0073
3.24	0.045
3.28	0.014
3.34	0.020
3.35	0.045
3.43	0.016
3.45	0.039
3.57	0.043
3.87	0.043
4.12	0.012
4.15	0.017
4.27	0.047
4.29	0.030
4.35	0.011
4.45	0.036
4.55	0.046
4.56	0.045
4.65	0.013
4.70	0.0062
5.64	0.036
5.72	0.010
5.77	0.043
5.88	0.043
5.96	0.021
6.01	0.034
6.27	0.021
6.32	0.0038
6.39	0.042

6.43	0.021
6.45	0.031
6.48	0.016
6.76	0.024
6.89	0.021
7.09	0.040
7.16	0.043
7.19	0.021
7.26	0.025
7.47	0.0034
7.50	0.029
7.94	0.038
7.96	0.021
7.97	0.014
8.33	0.033
8.34	0.0065

Supplementary Table S3. NMR identified metabolites.

METABOLITE	HMDB ID	δ (ppm)
2-Hydroxybutyric acid	HMDB0000008	0.89; 1.64; 1.73; 3.99
2-Hydroxyphenylacetic acid	HMDB0000669	3.56; 6.93; 7.21
Hippuric acid	HMDB0000714	3.96; 7.54; 7.62; 7.82
N-Acetylneuraminic acid	HMDB0000230	2.01; 2.04; 3.50; 3.72; 3.75; 3.90;3.98; 4.02
Panthotenic acid	HMDB0000210	0.89; 2.40; 3.37; 3.42; 3.49; 3.97
Phosphoethanolamine	HMDB0000224	3.24; 4.01
Spermidine	HMDB0001257	1.46; 1.61; 2.60
Succinic acid	HMDB0000254	2.39

Supplementary Table S4. Experimental concentration of identified metabolites at different times from pre-surgery (P) to discharge (D). Concentration values are expressed in µM/mM Creatinine except for Phosphoehtanolamine whose concentration is shown as nM/mM Creatinine. p-value is the result of non-parametric Mann Withney test between CVS-C and CVS-AKI patient. Bold letters correspond to statistically significant differences (p<0.05)

	**P**			**6h**			**24h**			**48h**			**72h**			**D**		
	CV-C	CV-AKI	p-value	CV-C	CV-AKI	p-value	CV-C	CV-AKI	p-value	CV-C	CV-AKI	p-value	CV-C	CV-AKI	p-value	CV-C	CV-AKI	p-value
2OH-butyric acid	4.312 [3.739-4.886]	5.094 [4.436-5.752]	**0.0129**	12.99 [10.61-16.37]	14.44[10.01-18.88]	0.6144	9.440 [8.053-10.83]	7.477 [5.698-9.255]	0.5031	9.537 [7.485-11.59]	4.485 [3.493-5.476]	**<0.0001**	11.29 [5.768-16.81]	4.608 [3.485-5.732]	0.6396	4.379 [3.599-5.159]	5.344 [4.435-6.253]	**0.0077**
2OH-phenylacetic acid	0.03087 [0.02547-0.03627]	0.04412 [0.03487-0.05336]	**0.0020**	0.005708 [0.003614-0.007802]	0.01061 [0.006129-0.01509]	0.1124	0.004752 [0.0007379-0.008765]	0.002666 [0.001562-0.003769]	**0.0104**	0.002726 [0.001622-0.003830]	0.001899 [0.0003563-0.003442]	0.6042	0.004245 [0.003076-0.005414]	0.003884 [0.001848-0.005919]	**0.0168**	0.02380 [0.01904-0.02856]	0.02799 [0.01612-0.03985]	0.4028
Hippuric acid	24.60 [19.82-29.38]	33.31 [24.17-42.44]	**0.0265**	6.364 [4.462-8.266]	8.123 [5.210-11.04]	**0.0167**	1.282 [1.018-1.547]	2.243 [1.110-3.375]	0.1670	2.413 [1.738-3.089]	3.985 [1.219-6.752]	0.3925	6.481 [4.495-8.467]	3.032 [2.293-3.770]	0.0891	16.99 [13.10-20.88]	15.51 [8.929-22.10]	0.0995
N-acetylneuraminic acid	0.04131 [0.03664-0.04598]	0.05038 [0.04153-0.05923]	0.0802	0.05453 [0.5021-0.05886]	0.07662 [0.06477-0.08848]	**0.0081**	0.04126 [0.03720-0.04532]	0.06208 [0.04872-0.07544]	**0.0103**	0.05411 [0.04696-0.06125]	0.06896 [0.05622-0.08171]	**0.0498**	0.07287 [0.05260-0.09314]	0.06991 [0.05660-0.08322]	0.1438	0.08255 [0.07066-0.09444]	0.0888 [0.06701-0.1108]	0.7524
Pantothenic acid	0.07006 [0.05581-0.08431]	0.1142 [0.07919-0.1492]	0.0682	0.01668 [0.01234-0.02102]	0.02586 [0.01772-0.03395]	**0.0458**	0.02176 [0.01594-0.02759]	0.01613 [0.006275-0.02596]	**0.0196**	0.03293 [0.02632-0.03954]	0.02026 [0.01161-0.02891]	**0.0010**	0.02414 [0.01308-0.03521]	0.008557 [0.004525-0.01260]	**0.0036**	0.04273 [0.02569-0.05977]	0.06402 [0.03423-0.09381]	**0.0020**
Phosphoethanolamine	13.54 [10.41-16.66]	17.53 [12.02-23.03]	**0.0226**	19.77 [15.14-24.41]	20.68 [13.49-27.87]	0.1939	12.54 [9.918-15.15]	13.09 [8.632-17.56]	0.9845	13.47 [9.564-17.39]	8.738 [5.292-12.18]	**0.0138**	9.747 [6.615-12.88]	10.15 [5.134-15.16]	0.5156	17.44 [11.61-23.27]	28.72 [9.553-47.89]	0.1382
Spermidine	0.02752 [0.02423-0.03081]	0.09407 [0.07706-0.1111]	**<0.0001**	0.1764 [0.1509-0.2019]	0.2495 [0.1874-0.3117]	**0.0115**	0.1409 [0.1288-0.1531]	0.1339 [0.1144-0.1533]	0.0925	0.1459 [0.1305-0.1612]	0.1080 [0.09360-0.1224]	**0.0007**	0.1288 [0.1038-0.1538]	0.09524 [0.08504-0.1055]	0.1617	0.08774 [0.07639-0.09909]	0.1023 [0-07188-0.1328]	0.2157
Succinic acid	1.602 [1.336-1.869]	2.183 [1.516-2.850]	0.2450	3.214 [2.624-3.803]	3.460 [2.414-4.505]	0.4130	2.415 [2.092-2.737]	0.2945 [0.1673-0.4218]	**<0.0001**	1.998 [1.687-2.309]	0.3936 [0.02042-0.7667]	**<0.0001**	1.771 [1.448-2.093]	1.324 [1.017-1.631]	0.0615	2.697 [1.872-3.521]	2.556 [1.448-3.664]	0.1151

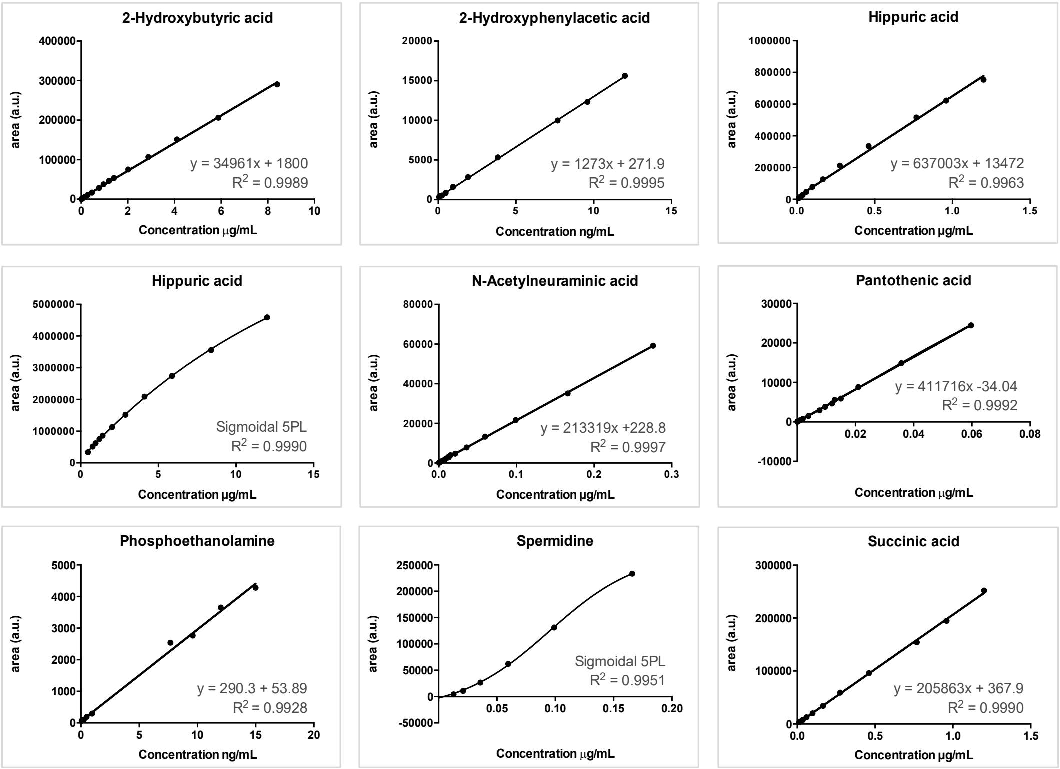

Supplementary Figure S1. Metabolites were quantified by SRM-LC-MS/MS. Equations for the lineal fit of each metabolite (or fit applied), concentration ranges and the square of the coefficient values (R2) are shown

METABOLITES

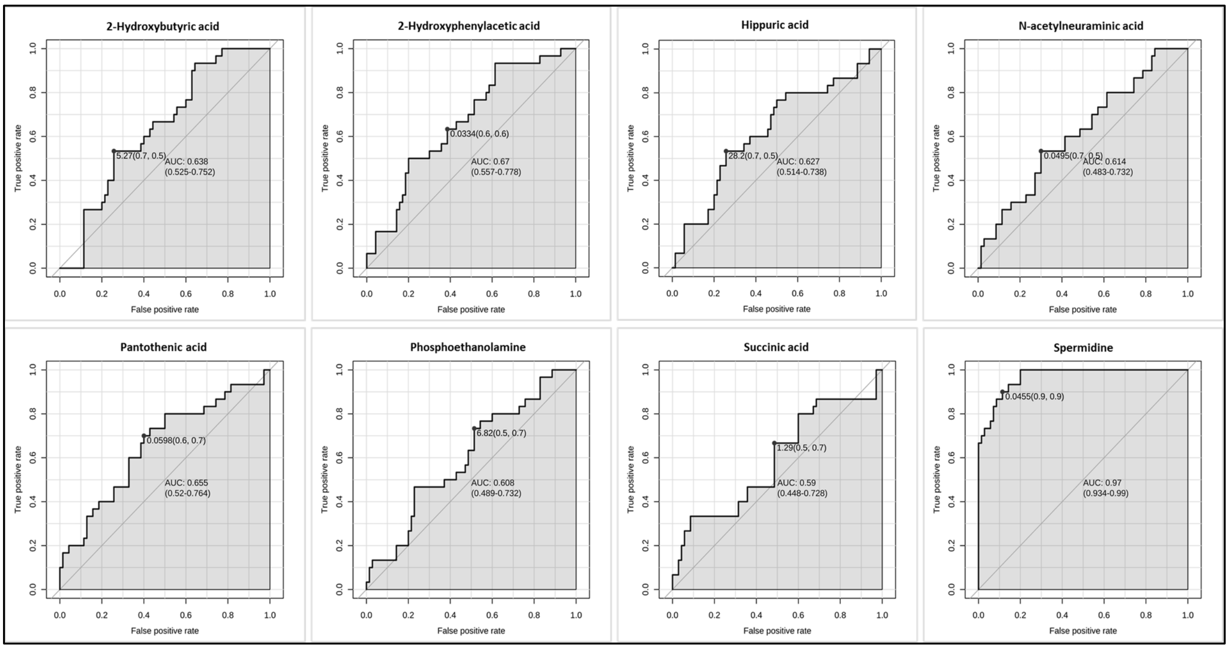

PROTEINS

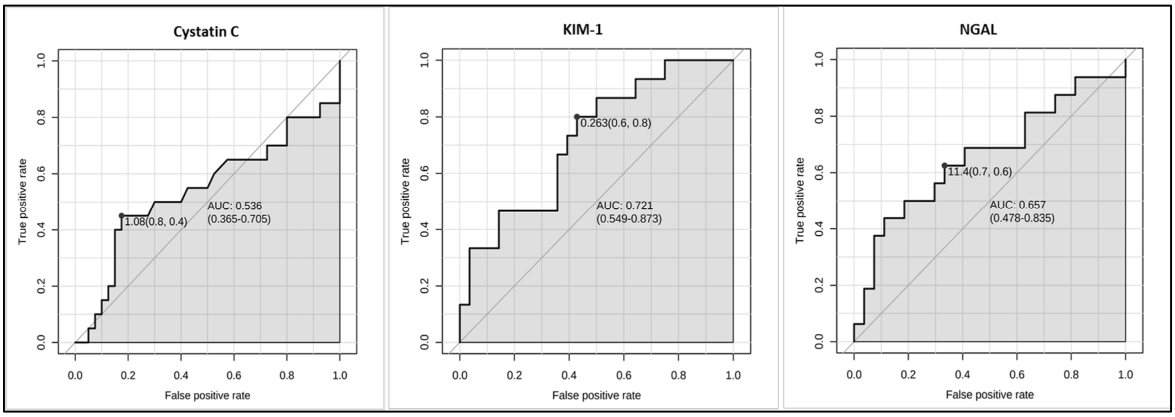

CLINICAL PARAMETERS

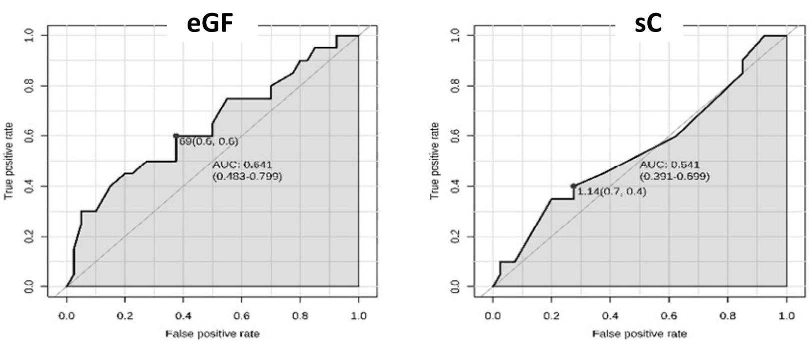

Supplementary Figure S2. Individual Receiving Operating Curves (ROC) for Metabolites, Proteins and Clinical parameters. The images show the Area Under the Curve (AUC) and the optimal cutoff at closest to top-left corner values. Both expressed with 95% IC
